# Deceptive digits: squamous cell carcinoma of the nail bed masquerading as glomus tumor

**DOI:** 10.1093/jscr/rjaf552

**Published:** 2025-07-25

**Authors:** Nishita Maheshwari, Mohit Vardey, Prashant Moon, Gokul Jorwekar

**Affiliations:** Department of General Surgery, DBVP Rural Medical College, Loni, Ahmednagar- 413736, Maharashtra, India; Department of General Surgery, DBVP Rural Medical College, Loni, Ahmednagar- 413736, Maharashtra, India; Department of General Surgery, DBVP Rural Medical College, Loni, Ahmednagar- 413736, Maharashtra, India; Department of General Surgery, DBVP Rural Medical College, Loni, Ahmednagar- 413736, Maharashtra, India

**Keywords:** nail bed malignancy, delayed diagnosis, glomus tumor

## Abstract

Subungual squamous cell carcinoma is a rare malignancy that can mimic benign nail conditions, leading to delayed diagnosis and treatment. We present a case of a 50-year-old male with a 10-year history of recurrent nail bed growth of the left thumb initially diagnosed as a glomus tumor based on clinical and radiological findings. Despite multiple prior surgical interventions, the patient experienced recurrence of the lesion. Final histopathological examination revealed squamous cell carcinoma, necessitating thumb amputation and axillary lymph node clearance. This case highlights the importance of maintaining a high index of suspicion for malignancy in recurrent nail bed lesions and the critical role of histopathological confirmation in nail bed tumors.

## Introduction

Squamous cell carcinoma arising from the nail bed is uncommon. These malignancies often present diagnostic challenges as they can mimic various benign conditions, including glomus tumors, pyogenic granulomas, or chronic paronychia [1]. The diagnostic challenge is compounded by the relatively indolent course of subungual SCC, with patients often presenting after multiple failed treatments for presumed benign pathology. Delayed diagnosis can lead to local tissue destruction, metastasis, and increased morbidity [2, 3]. This case report describes a patient with a long-standing history of recurrent nail bed growth, ultimately diagnosed as squamous cell carcinoma.

## Case presentation

**Figure 1:**
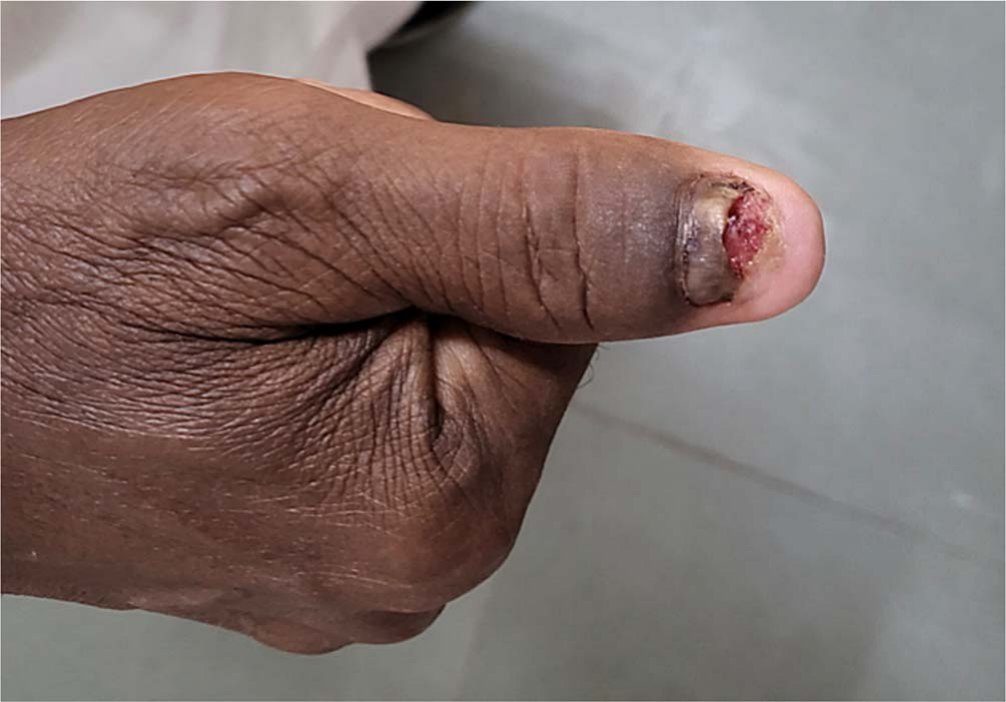
Recurrent lesion over left thumb.

A 50-year-old male presented to our surgical clinic with complaints of a recurrent growth over the left thumb persisting for ~10 years. The patient had undergone multiple nail removal procedures during this period, but experienced consistent recurrence of the lesion (Fig. 1). His medical history was significant for well-controlled diabetes mellitus with a 15-year history of the condition. He was a non-smoker with no prior history of skin cancer.

On physical examination, the distal nail of the left thumb was broken off, and a 2 × 1 cm growth was observed over the remaining nail. The lesion was tender to palpation with minimal bleeding. No lymphadenopathy was detected in the left axilla on initial examination. Initial differential diagnoses included glomus tumor, subungual exostosis, and chronic paronychia.

### Diagnostic assessment

Magnetic resonance imaging of the left thumb revealed an ulceroproliferative, homogeneously enhancing solid lesion measuring 0.6 × 1.25 × 1.9 cm in the subungual region of the left thumb. T1-weighted images showed intermediate signal intensity while T2-weighted images demonstrated high signal intensity. Post-contrast sequences showed homogeneous enhancement with no evidence of bone invasion. Ultrasonography demonstrated intense vascularity within the lesion. Based on these clinical and radiological findings, a preliminary diagnosis of glomus tumor was made.

Standard laboratory investigations, including complete blood count, liver and renal function tests, and coagulation profile, were within normal limits. Chest radiography showed no evidence of metastatic disease.

### Therapeutic intervention

**Figure 2:**
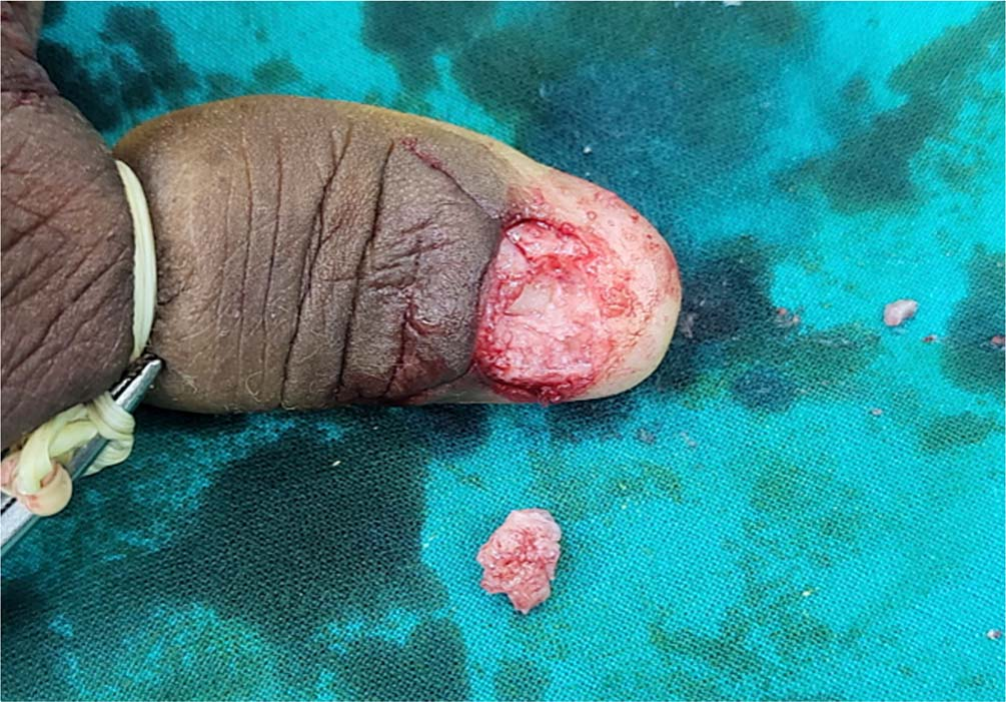
Intra-operative image after excision of lesion.

**Figure 3:**
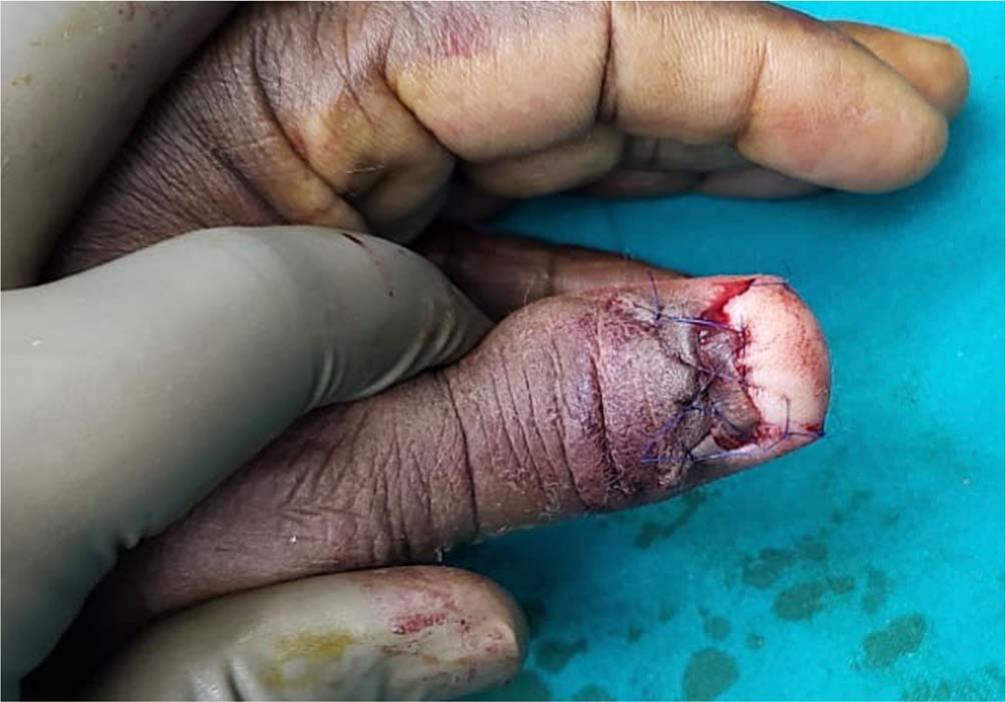
Reconstruction with V-Y advancement flap.

The patient underwent excision of the tumor. The nail plate was completely removed, exposing a friable, vascular mass attached to the nail bed. Careful dissection was performed to excise the tumor with a 2-mm margin of surrounding nail bed (Fig. 2). A V-Y advancement flap was utilized for reconstruction of the nail bed defect (Fig. 3). The excised specimen was sent for histopathological examination.

### Pathological findings

Contrary to the clinical suspicion of a glomus tumor, histopathological examination revealed a well-differentiated squamous cell carcinoma, with focal keratinization and invasion of the underlying dermis. The tumor cells showed moderate pleomorphism, increased nuclear-to-cytoplasmic ratio, and numerous mitotic figures. The surgical margins were positive at the deep margin. This unexpected finding necessitated more aggressive surgical management.

### Follow-up

Following the histopathological diagnosis of squamous cell carcinoma, the patient underwent left thumb amputation with left axillary lymph node clearance. Postoperative recovery was uneventful. The patient was referred to medical oncology for consideration of adjuvant therapy.

## Discussion

This case illustrates several important clinical points. First, it highlights the challenging nature of diagnosing subungual malignancies. The patient's 10-year history of recurrent growth with multiple prior nail removal procedures represents a significant delay in definitive diagnosis and appropriate treatment. Second, it demonstrates the limitations of relying solely on clinical and radiological findings for definitive diagnosis of nail bed lesions.

Subungual SCC is often misdiagnosed as a benign condition due to its indolent course and nonspecific presentation. Common misdiagnoses include fungal infection, pyogenic granuloma, glomus tumor, or verruca vulgaris [1, 4]. The chronic nature of these lesions and their tendency to recur after conservative management should raise suspicion for malignancy.

In retrospect, several factors in this case could have raised suspicion for malignancy earlier, including the recurrent nature of the lesion despite multiple interventions and the chronicity of symptoms. This underscores the importance of maintaining a high index of suspicion for malignancy in any recurrent nail bed lesion and obtaining histopathological confirmation early in the disease course [5, 6].

## Conclusion

SCC represents a diagnostic challenge due to its rarity and ability to mimic benign conditions. This case emphasizes the importance of histopathological examination of all excised nail bed lesions, especially those with a history of recurrence despite appropriate treatment. Early diagnosis and appropriate surgical management are essential for optimal outcomes in patients with subungual SCC [5].
